# In vivo effect of magnetic microspheres loaded with E2-a in the treatment of alveolar echinococcosis

**DOI:** 10.1038/s41598-020-69484-z

**Published:** 2020-07-28

**Authors:** Zhi Li, Guochao Zhang, Yanping Luo, Qi Gao, Jianghua Wang, Chong Chen, Xiaoying Xu, Yingying Zhao, Tingting Li, Xingming Ma

**Affiliations:** 1 Department of Immunology, School of Basic Medical Sciences, Lanzhou University, Lanzhou, 730000 China; 2 Key Lab of Preclinical Study for New Drugs of Gansu Province, Lanzhou, 730000 China; 3 The Second Hospital of Lanzhou University, Lanzhou, 730030 China; 4 Department of Clinical Laboratory, Xi’an Ninth Hospital, Xi’an, 710054 China; 5 Institute of Pathology, School of Basic Medical Sciences, Lanzhou University, Lanzhou, 730000 China

**Keywords:** Biotechnology, Diseases, Nanoscience and technology

## Abstract

The alveolar echinococcosis of human is a severe helminthic disease caused by the larva of *Echinococcus multilocularis* tapeworms. Novel compounds or therapy strategies for the treatment of alveolar echinococcosis are urgently needed due to the limitation of the widely used albendazole. Magnetic microspheres as drug carriers in magnetically targeted therapy of tumor have gained growing interests advantaged by delivering the drug to the aimed site, achieving localized therapeutic effect effectively under the influence of an external magnetic field. In this study, we formulated magnetic microspheres loaded with E2-a (PLGA-Fe-E2-a) and identified the activity in *E. multilocularis*-infected mice which infected with 3,000 protoscoleces intraperitoneally. Compared with the untreated control, with the help of a magnet, there was a significant reduction in parasite burden with PLGA-Fe-E2-a treatment and similar reduction observed with albendazole. PLGA-Fe-E2-a treatment group also showed a significant increase in the IFN-γ level and impaired morphological and ultrastructural alterations. Most importantly, one-third concentrations of E2-a from PLGA-Fe-E2 based on the release profile of E2-a was equally effective in inhibiting metacestode growth as E2-a treated group, supporting efficacy and bioavailability of a drug. It will be an alternative treatment for alveolar echinococcosis using magnetic microspheres as drug carriers.

## Introduction

Alveolar echinococcosis (AE) is caused by the larval stage (metacestodes) of *Echinococcus multilocularis* (*E. multilocularis*), which is a neglected and highly lethal zoonotic helminthic disease if treated inappropriately^1^. AE grow aggressively and continue to infiltrate the host tissues in a tumor-like behavior, which reduce the possibility of radical excision of the parasitic lesions. The benzimidazole derivatives i.e., albendazole (ABZ) and mebendazole are still mainstay of the current chemotherapy against AE, although they act parasitostaticlly. Alternative options against AE such as modifying the existing drugs based on nanostructured or microstructured entities, developing the conventional anti-infection or anti-tumor reagents for anti-hydatid application or attempting combinated therapy instead of single strategy have been attempted^2–4^.

With the aid of a magnetic field, magnetical targeted drug-delivery systems provide an innovative chemotherapeutic option, which can delivery drugs directly to the desired target areas, fix them at a local site avoiding rapid clearance by the reticuloendothelial system and minimize systemic toxicity^5^. Due to the above properties, magnetic particles as drug carriers have been used to delivery agents such as doxorubicin, mitomycin C and sulforaphane for cancer therapy^6–8^. Recently, iron oxide mesoporous magnetic microparticles as novel carriers have been reported to have promising results against cancer due to their biocompatibility, effective drug delivery, high-level drug accumulation in the target tissue and deep tumor penetration^9^. The iron oxide magnetic particles may offer a platform to treat *E. multilocularis* metacestodes owing to these similarities between *E. multilocularis* metacestodes and malignant tumors^10^. However, to our knowledge, there are few studies on the application of magnetic particles against AE.

The seeds of *Sophora moorcrofiana* (*S. moorcrofiana*), which have been used as traditional Tibetan medicine to treat parasitoses in China, possess antimicrobial, antitumor, immunoregulatory and antiparasitic pharmacological activities^11–15^. E2-a is a water-soluble alkaloid and an active ingredient of *S. moorcrofiana* seeds. E2-a (≥ 0.05 mg/ml) is highly effective in killing the protoscoleces of *Echinococcus granulosus* (*E. granulosus*) in vitro^15^. However, E2-a shows weaker protoscolicidal effects when compared to albendazole in protoscolex-infected mice^15,16^.

One important reason for this discrepancy between in vitro and in vivo activity is the low bioavailability of E2-a. The matrine and sophocarpinehe as the main ingredients of E2-a have been proved to have poor oral bioavailability^15,17,18^. Therefore, it is essential to use optimized delivery systems or novel approaches to improve the bioavailability of E2-a.

Magnetite (Fe_3_O_4_) has been approved for biomedical and medical applications due to its potential values such as strong magnetic property, low toxicity and good biocompatibility. Poly (d,l-lactic-co-glycolic) (PLGA) due to its favorable features such as excellent biodegradability, administration safely and well-documented biocompatibility has been widely used in biomedical field.

Therefore, we used the above materials to formulate the magnetic microspheres loaded with E2-a (PLGA-Fe-E2-a) using water-in-oil-in-water (W/O/W) emulsions method. And the treatment efficacy of PLGA-Fe-E2-a against murine alveolar echinococcosis was investigated.

## Results

### Physicochemical characteristics of PLGA-Fe-E2-a

**Figure 1:**
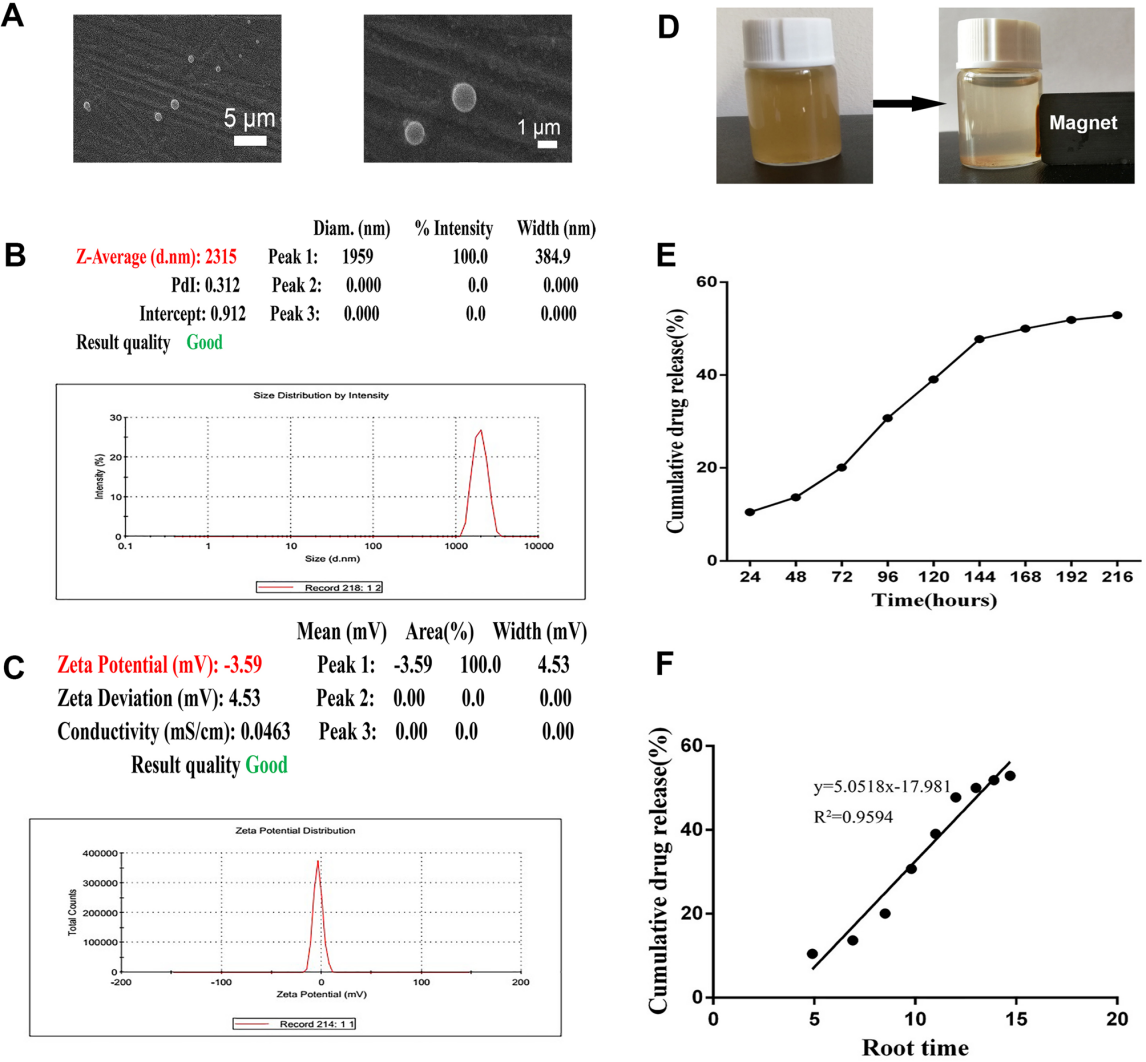
Physicochemical characteristics of magnetic microspheres loaded with E2-a (PLGA-Fe-E2-a). Representative SEM micrographs of the surface morphology of PLGA-Fe-E2-a. The bar of the panel was 1 μm (left) or 5 μm (right). The particle size (**C**) and zeta potential (**D**) of PLGA-Fe-E2-a were assessed by dynamic light scattering. The particle size and polydispersity index of PLGA-Fe-E2-a were 2,315 nm and 0.312, respectively. The zeta potential of PLGA-Fe-E2-a was − 3.50 mV. PLGA-Fe-E2-a dispersed in the water (left) and in the presence of an external magnet (right) (**D**). In vitro cumulative release profile of PLGA-Fe-E2-a at 37 °C and Higuchi analysis of E2-a from PLGA-Fe-E2-a (**E**,**F**). Notes: Higuchi analysis was carried out by plotting square root time against percent drug release. E2-a concentrations were measured by a UV–visible spectrophotometer at 210 nm.

The PLGA-Fe-E2-a, magnetic microspheres loaded E2-a, were spherical with comparatively smooth surfaces evidenced by scanning electron micrographs (SEM) (Fig. 1A). In addition, the PLGA-Fe-E2-a showed a monomodal population at diameter of 2.315 µm with polydispersity index (PDI) of 0.312 and zeta potential of − 3.59 mV, respectively (Fig. 1B,C). As shown in Fig. 1D, the aggregation of PLGA-Fe-E2-a from aqueous solution in the presence of a magnet showed the magnetic property of PLGA-Fe-E2-a. The encapsulation efficiency and drug loading of PLGA-Fe-E2-a were 58.1% and 4.3% respectively (data not show). Based on their goodness of fitting, zero order and Higuchi model were selected to identify the profiles of cumulative release of E2-a from PLGA-Fe-E2-a. The liberation of E2-a from the PLGA-Fe-E2-a was calculated from calibration curves (y = 28.703x + 0.03573) with correlation coefficient of 0.9982 using the zero order model. The liberation of E2-a from PLGA-Fe-E2-a exhibited a small burst release in the first 24 h and then slow release constantly. It could be seen that 20% of the drug released at 72 h (Fig. 1E). In Higuchi model, the plots showed a high linearity with regression value of 0.9594 (Fig. 1F).

### PLGA-Fe-E2-a treatment reduced the parasite burden

The efficacy of PLGA-Fe-E2-a on *E. multilocularis* infection was assessed*.* The wet weight of metacestodes reduced  significantly in abendazole (ABZ), PLGA-Fe-E2-a or E2-a treated group when compared to the untreated control group, respectively (2.59 ± 0.6 g, 2.72 ± 0.52 g and 3.57 ± 0.57 g versus 5.12 ± 1.51 g, *p* < 0.05) (Table 1). However, the parasite burden had no significant difference in PLGA-Fe-E2-a, ABZ and E2-a treated group (*p* > 0.05), suggesting their similar effects on the growth of the metacestodes in vivo.

**Table 1:** Comparative distribution of the parasite and plasma concentration profiles of IFN-γ in *Echinococcus multilocularis-*infected mice treated with albendazole (ABZ) (100 mg/kg/day), E2-a (50 mg/kg/day) or PLGA-Fe-E2-a (50 mg/kg/3 day).

Groups	Weight of cysts (g)	Parasite inhibition rate (%)	IFN-γ (pg/ml)	TNF-β (pg/ml)
Untreated control	4.91 ± 1.48	–	1,355.62 ± 248.97	304.69 ± 312.47
Albendazole	2.50 ± 0.63***	49.2	1,479.88 ± 58.28	381.03 ± 158.01
E2-a	3.57 ± 0.57*	27.4	1625.86 ± 178.14*	167.41 ± 80.50
PLGA-Fe-E2-a	2.39 ± 1.15***	51.4	1732.90 ± 136.52*	452.00 ± 412.85

The weight of cysts and levels of IFN-γ and TGF-β were measured (n = 6). All data were analyzed by using SPSS (version 19.0). The data were analyzed by one-way analysis of variance (ANOVA). Data were expressed as mean ± standard deviation (SD) for two independent experiments.

*p < 0.05 and ***p < 0.001 versus untreated control group.

### PLGA-Fe-E2-a treatment induced severe morphological and ultrastructural changes in metacestodes

**Figure 2:**
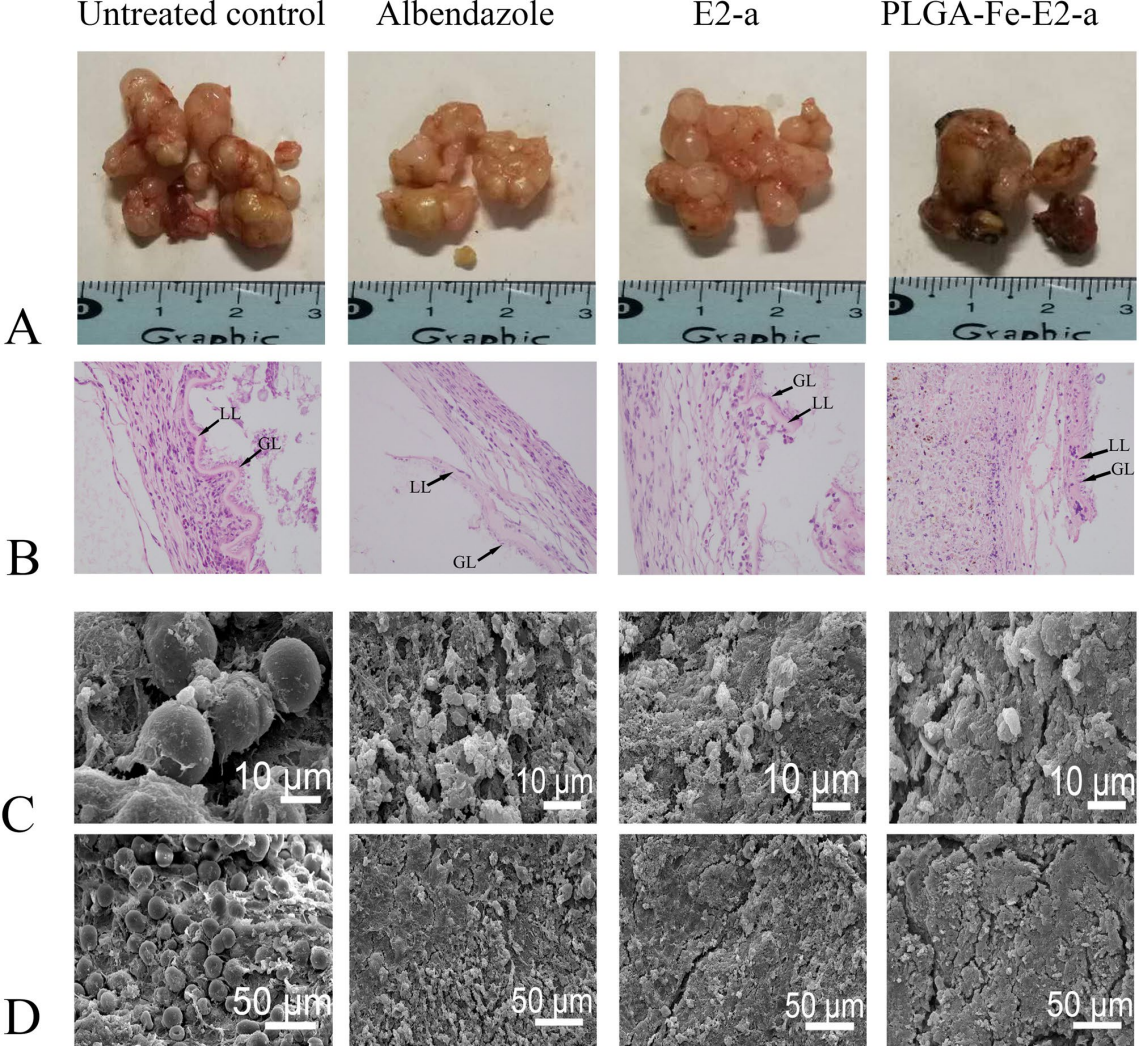
In vivo treatment of *E. multilocularis* infected mice with PLGA-Fe-E2-a. BALB/c mice were intraperitoneally infected with *E. multilocularis* metacestodes. At the 14th week after infection, mice with secondary alveolar echinococcosis were treated with PBS, albendazole (100 mg/kg/day), E2-a (50 mg/kg/day) or PLGA-Fe-E2-a (50 mg/kg/3 day). After six weeks of treatment, mice were sacrificed. The parasite cysts were resected and weighted. Macroscopic (**A**), the representative pathological characteristics (H&E stain, magnification 100 ×). Samples of parasite vesicles were processed for haematoxylin and eosin (H&E) staining (**B**). Samples of cysts were used to scanning electron microscopy (SEM) examination (**C**,**D**). Note the distorted morphology of the germinal layer (GL) in those specimens treated with PLGA-Fe-E2-a. *GC* germinal layer, *LL* laminated layer. Bar = 10 μm (**C**) or 50 μm (**D**).

In the untreated control group, the metacestodes presented a larger number of small vesicles and more blood vessels compared with that in the ABZ and PLGA-Fe-E2-a treated groups (Fig. 2A). An intact consecutive germinal layer and a hyaline membrane with uniform thickness (laminated layer) were observed in the untreated control group (Fig. 2B). At the ultrastructural level, it was noted that the cysts from the untreated control group appeared intact and densely packed with morphologically different cells (Fig. 2C,D).

In the PLGA-Fe-E2-a group, the larval cysts appeared dark brown, conglomerate and suppressed blood vessels (Fig. 2A). Cysts isolated from PLGA-Fe-E2-a group lacked the typical structure. An irregularly swollen laminated layer and a partially dissolved germinal layer cells were observed microscopically (Fig. 2B). The germinal layer of metacestodes from PLGA-Fe-E2-a treated group lost the multicellular structures and featured with cellular debris by SEM (Fig. 2C,D).

In the ABZ group, we observed that the metacestodes were conglomerated with the swollen laminated layer and reduced cells of the germinal layer (Fig. 2).

### PLGA-Fe-E2-a treatment increased the expression of IFN-γ

In parasite infection, cytokines play a key role and the Th1 cytokines are connected with parasite growth restriction and clearance^15,19^. However, Th2-oriented cytokines resulted in metacestode growth at the later stage of infection. To investigate the role of PLGA-Fe-E2-a orchestrating the immune response, the expression profiles of TGF-β selected Th2 cytokine and IFN-γ Th1-oriented cytokine in mice infected with *E. multilocularis* metacestodes were analyzed. Low concentrations of IFN-γ were present in the untreated control group. The level of IFN-γ had a significant increase in PLGA-Fe-E2-a group compared to the untreated control group (*p* < 0.05). There was no statistical difference in the level of TGF-β in all groups (*p* > 0.05) (Table 1).

## Discussion

The magnetic microspheres loaded with E2-a were formulated using a modified W/O/W emulsions method. PDI, the width distribution of particles, was scaled from 0 (perfectly uniform) to 1 (polydisperse with multiple particle size). The PDI of microspheres less than 0.5 were considered to be monodisperse and acceptable for delivering drugs^20^. The PDI of PLGA-Fe-E2-a was 0.312, which indicated that PLGA-Fe-E2-a were suitable for drug delivery.

The release behaviors of drugs from microspheres were influenced by several factors, such as the preparation temperature, the size of microspheres, the polymer concentration and molecular masses^21^. The liberation of E2-a from PLGA-Fe-E2-a exhibited a small burst release in the first 24 h and then a slow release. It could be seen that 20% of the drug was released at 72 h.

The size of PLGA-Fe-E2-a was 2.3 µm in diameter, which limited them cross the endothelial barrier^22^. Besides, limited by the strength and penetration depth of the magnetic field, magnetic targeted drug-delivery systems were suitable for the superficial or intracavitary therapy^23^. The secondly AE infection model was based on intraperitoneal inoculation of metacestodes, and thus the parasites grown primarily in the peritoneal cavity^4^. Intraperitoneal application of drugs may be a useful effort to treat the parasites. Hemphill found that intraperitoneal application of mefloquine and DB1127 effectively reduced the parasite weight in vivo rodent models^10^. Thus, in this study, PLGA-Fe-E2-a were attempted to administrate intraperitoneally and to evaluate their potential treatment effects.

The treatment efficacy was evaluated by the mean cysts weight, immune responses, morphological and ultrastructural changes of cysts. In this research, ABZ was used as a positive control. There was a significant reduction in parasite burden with PLGA-Fe-E2-a treatment and similar reduction observed with widely used ABZ.

The parasite burden had no significant difference in PLGA-Fe-E2-a and E2-a treated group (*p* > 0.05). In other words, one-third concentrations of E2-a from PLGA-Fe-E2 based on the release profile of E2-a was equally effective in inhibiting metacestode growth as E2-a treated group, supporting efficacy and bioavailability of a drug. The metacestodes of AE, fluid-filled vesicles, were composed of an inner germinal layer and an outer laminated layer. ABZ reduced the germinal layer cells of metacestodes. Our results agreed with a previous study which found ABZ was active against the germinal layer cells of *E. mutilocularis*^24^. The loosened laminated layers were found in E2-a or PLGA-Fe-E2-a treated group at morphological levels, however, further research was needed to distinguish the loose structure caused by drugs or sample preparation. Besides, the lost multicellular structures of the germinal layers were found in E2-a and PLGA-Fe-E2-a group at the ultrastructural level. Moreover, the cell debris in the germinal layer were found in PLGA-Fe-E2-a treated group. To our knowledge, this is first time to describe the effect of E2-a or PLGA-Fe-E2-a on the germinal layer cells of alveolar hydatid cyst.

Th1-oriented cytokines played an important role in parasite clearance and growth restriction. However, Th2-oriented cytokines resulted in metacestode growth. During the later stages of AE infection, the initial expression of Th1-oriented cytokines was gradually replaced by Th2 lineages^15,20,25^. PLGA-Fe-E2-a treatment induced a significant reduction in parasite burden accompanying increased IFN-γ secretion. The increased IFN-γ levels may be an important indicator of the effectiveness of drug at the late phase of infection of *E. multilocularis*.

Magnetic microspheres as carriers of drug delivery systems had gained growing interests advantaged by delivering drug to the specific site and achieving effective localized therapeutic effect associated with the elevated local drug concentrations under an external magnetic field^26^. PLGA-Fe-E2-a resulted in a better efficacy that may attribute to its improved bioavailability of drugs and targeted delivery by means of physical force from magnetic fields. The enhanced treatment efficacy of PLGA-Fe-E2-a may also be connected with the features of magnetic microspheres that can avoid the rapid clearance by reticuloendothelial system^27^. Also, the magnetic drug-containing microspheres consist of a magnetic core and polymeric shell that can protect drugs from degradation, enhance drug stability and sustained-release^28^. All the results strongly suggest that PLGA-Fe-E2-a were a promising candidate strategy for the effective treatment of echinococcosis.

Our data indicated that PLGA-Fe-E2-a improved the bioavailability of E2-a after enclosed magnetic microspheres and showed the same protoscolicidal effects as abendazole in *E. multilocularis*-infected mice. In further research, some pharmacokinetic and pharmacodynamic properties such as in vivo release and the mechanism of action of PLGA-Fe-E2-a will be elucidated and identified. And it will be interesting to study dose response and time course and administration route to enhance the efficacy of PLGA-Fe-E2-a.

## Conclusion

In conclusion, PLGA-Fe-E2-a were suitable for drug delivery and exhibited a lethality activity on *E. multilocularis* by accelerating IFN-γ secretion and inducing severely morphological and ultrastructural damages in cysts. The present study firstly formulated and demonstrated that PLGA-Fe-E2-a may be as a new interesting and promising therapeutical strategy for the treatment of *E. multilocularis* infection.

## Materials and methods

E2-a, a water-soluble alkaloid extracted from *S. moorcrofiana* seeds, was kindly provided by Yanping Luo. Albendazole and PLGA were purchased from Sigma (St. Louis, Missouri, USA). Poly (vinyl alcohol) (PVA) was obtained from BBI life sciences. Nano-sized Fe_3_O_4_ particles was provided by Miao-miao Yuan, Lanzhou University. ELISA kits were purchased from (MultiSciences (Lianke) Biotech, China). All purchased chemicals were used without further purification.

### Preparation of the alkaloid E2-a extract

The alkaloid E2-a extract was prepared as described previously^14^. After being sieved using a 10-mesh steel strainer, the pulverized *S. moorcrofiana* seeds were soaked in 60% ethanol for 24 h subsequent to extract at 80 °C for 4 h by refluxing extraction. Then the above extract was concentrated using a rotary evaporator. The fluid extract was collected and adjusted to a pH of 4 with 12 M HCl. Subsequently, the acidulated fluid was centrifuged at 2,000 g for 10 min. The supernatant was collected and adjusted to a pH of 12 with 10 M NaOH. Then the supernatant liquor contained the water-soluble alkaloidal fraction was collected and extracted with chloroform. The dry material was obtained after removal of chloroform and separated by silica gel column chromatography. Finally, the alkaloid E2-a extract with low molecular polarity (main band Rf = 0.93) were obtained. The thin layer chromatography and the high performance liquid chromatography indicated that matrine (43%) and sophocarpine (26%) were major components of E2-a.

### Preparation of magnetic microspheres loaded with E2-a

The PLGA-Fe-E2-a were formulated using the W/O/W emulsions previously described with modification^29^.

In the first step the primary water-in-oil emulsions were prepared^29^. PLGA (1% w/v) solution was previously solubilized in dichloromethane and sonicated until dissolved thoroughly. The PLGA solution was used as the oil phase. The internal aqueous phase consisted of 20 mg of Fe_3_O_4_ nanoparticles and E2-a. The Fe3O4 nanoparticles were synthesized using the chemical coprecipitation method as described^30^. The 2.5 ml internal aqueous phase together with 4 ml oil phase were then mixed and sonicated using an ultrasonic homogenizer (Ningbo Scientz Biotechnology Co., Ltd., time 60 s, pulse sequence 0.1 son and 0.1 s off, ice bath). Subsequently, in the second step, the primary emulsions were added drop-wise over the external aqueous phase under electromagnetically stirring. The external aqueous phase contained 50 ml 2% (w/v) PVA. The final mixture was sonicated for 2 min and stirred at 2,000 g for 24 h until the whole organic solvent and water were evaporated and microspheres were formed. The microspheres were collected by centrifugation and washed several times with distilled water to remove the non-encapsulated drugs and the residual drugs adsorbed on the surface of the microspheres. Then the microspheres were isolated with centrifuge membrane filters (0.45 µm) and dried under vacuum at room temperature in the dark.

### Physicochemical characteristics of PLGA-Fe-E2-a

The mean particle size, polydispersity index (PDI) and zeta potential of PLGA-Fe-E2-a were assessed using Zetasizer Nano ZS ZEN 3,600 (Malvern Instruments Ltd, Malvern, UK). The micrographs of PLGA-Fe-E2-a mounted on aluminum stubs with gold coated were performed at an accelerating voltage of 15–20 kV using scanning electron microscope (Model JSM-5600LV, Japan Electron Optics Laboratory Co. Ltd. Japan).

### Encapsulation efficiency and drug loading

The encapsulation efficiency and drug loading of PLGA-Fe-E2-a were conducted in a modified method^6^. Briefly, PLGA-Fe-E2-a (10 mg) were suspended in 10 ml acetonitrile and stirred continuously. Then the precipitated pellets were retained, dried and re-dissolved with 1 ml PBS. The concentration of E2-a in the PBS was determined by UV–Vis spectroscopy at 210 nm.

- % Encapsulation efficiency = weight of encapsulated drug/weight of initial drug loading in the solution × 100
- % Drug loading = weight of encapsulated drug/weight of microspheres × 100

### Drug release

In vitro release profile of E2-a from PLGA-Fe-E2-a was performed as described^6^ and in short was as follows: The dried microspheres (10 mg) were transferred to a conical flask and suspended in 40 ml of PBS buffer (pH 7.4). The conical flask was shaken in a shaker incubator (100 rpm) at 37 °C. At scheduled times (10 h, 24 h, 48 h, 72 h, 96 h, 120 h, 144 h, 168 h, 192 h, 216 h and 240 h), 2 ml aliquots were taken from each conical flask for UV–Vis measurements and then added the same volume of PBS buffer. Bipartite aliquots were run for each time interval. The zero order (cumulative amount of drug released vs time) and Higuchi kinetic model (cumulative percentage of drug released vs square root of time) were performed as described^31^.

### Animals

Specific pathogen free (SPF) female BALB/c mice, aged between 6 and 8 weeks, were purchased from the Laboratory Animal Center of Lanzhou University. The mice were maintained in laboratory conditions with 12 h light/dark cycle and controlled temperature (22 ± 1 °C) and were received food and water ad libitum. All experiments were approved by the Institutional Animal Care and Use Committee of Lanzhou University and carried out in accordance with the local ethics committee rules.

### Efficacy in a murine model of AE

The preparation of the *E. multilocularis* metacestodes and the experimental infection were established as described^32^. Each mouse was injected with 3,000 protoscoleces intraperitoneally. According to our previous experimental results, the *E. multilocullaris*-infected mice would be constructed at the 14th week after infection. Besides, the autopsy results of five randomly selected mice also confirmed that the mice were infected with *E. multilocularis* successfully. Then the infected mice were randomly assigned into four groups (6 mice/group) with consecutive treatment for 6 weeks. (1) untreated control group: mice were received 0.2 ml/day PBS intraperitoneally; (2) albendazole group: mice were administered 100 mg/kg/day albendazole by gavage; (3) E2-a group: mice were received intraperitoneal injection E2-a 50 mg/kg/day; (4) PLGA-Fe-E2-a group: mice were injected PLGA-Fe-E2-a 50 mg/kg/3 day intraperitoneally with a magnet tied to the abdomen of mice for 30 min every 3 days. Mice were sacrificed by cervical dislocation followed by anesthetized with intraperitoneal injection of 0.3% pentobarbital sodium. The vesicular cysts were resected carefully, photographed and weighted. The efficacy was calculated according to the following formula:

$$\begin{aligned} \left( \% \right){\text{ of efficacy}} & = ({\text{the average wet weight of AE vesicles in the untreated}} \\ & \quad {\text{control group}} - {\text{the average wet weight of AE vesicles in}} \\ & \quad {\text{the treated group}})/{\text{the average wet weight of AE vesicles}} \\ & \quad {\text{in the untreated control group}} \times 100. \\ \end{aligned}$$

### Morphologic study

Samples of parasite vesicles were processed for haematoxylin and eosin (H&E) staining as described^16,33^ and observed under light microscopy. Samples of cysts were used to SEM examination. The SEM was performed as described^4,34^. Briefly, the specimens, isolated vesicles, were fixed in 2.5% glutaraldehyde solution at 4 °C. Subsequently, the samples were stained for 2 h in a 1% osmium tetroxide solution at 4 °C. After washed with PBS buffer, the samples were dehydrated with increasing concentrations of ethanol and then immersed in 2% isoamyl acetate for 20 min. Finally, the specimens were mounted on aluminum stubs and sputter-coated with gold and examined with a scanning electron microscope (Model JSM-5600LV, Japan Electron Optics Laboratory Co. Ltd. Japan).

### Serum concentrations of IFN-γ and TGF-β

After clotted at 37 °C for 30 min, the blood samples from sacrificed animals were centrifuged at 3,000 g for 15 min. The supernatant was collected and stored at − 80 °C until further analysis. The cytokine expression levels of IFN-γ and TGF-β were measured using sandwich enzyme-linked immunosorbent assays (MultiSciences, China) according to the manufacturer's instructions.

### Statistical analysis

Statistical analysis was analyzed using SPSS (version 19.0). One-way analysis of variance (ANOVA) of the Student Neuman-Keul’s procedure or nonparametric test of the Mann–Whitney U test were used. Data were shown as mean ± standard deviation (SD). *p* < 0.05 were considered to be significant differences. Data presented as scatter plots or bar charts were performed using GraphPad Prism version 6.00.

## Data Availability

The datasets during the current study are available from the corresponding author on reasonable request. And all submitted manuscripts are available.
